# Socioeconomic Status and COVID-19-Related Psychological Panic in China: The Role of Trust in Government and Authoritarian Personality

**DOI:** 10.3390/ijerph182010888

**Published:** 2021-10-16

**Authors:** Xiaona Xie, Tingting Wu, Yue Zhang, Yongyu Guo

**Affiliations:** School of Psychology, Nanjing Normal University, Nanjing 210097, China; xiexiaonaShirley@126.com (X.X.); 192302038@stu.njnu.edu.cn (T.W.); 182301007@stu.njnu.edu.cn (Y.Z.)

**Keywords:** socioeconomic status, psychological panic, trust in government, authoritarian personality, COVID-19

## Abstract

Although the health and economic risks of COVID-19 may differ for higher- and lower-socioeconomic-status (SES) populations, some studies found that people with lower SES do not necessarily experience more psychological panic. In this research, we examine how SES is related with psychological panic during the COVID-19 pandemic using a large nationwide Chinese sample. Participants were 933 adults (mean age = 30.04, SD = 8.19) who completed an online questionnaire between 11 and 12 February 2020. Lower SES individuals have higher trust in government and thus experience less psychological panic, and the indirect effect of this trust suppresses the direct negative association between SES and psychological panic. In addition to this difference in trust in government between lower- and higher-status individuals, the indirect effect of the trust only exists among people with low (not high) authoritarian personalities. This study provides evidence that political trust may serve as a buffer, suppressing the negative association between SES and psychological panic; thus, policies and actions enhancing political trust are vital to support the mental health of individuals with lower SES during the pandemic, especially for citizens with low authoritarian personalities.

## 1. Introduction

Since COVID-19 was first identified in Wuhan in December 2019, this pandemic has become a global public health crisis that has profoundly affected individuals and communities. Along with threats to physical health, COVID-19 also has a negative effect on mental health: from avoiding becoming ill and dying and coping with changes in normal life caused by lockdowns to enduring the trauma of losing loved ones [1,2,3]. One of the most prominent phenomena of mental distress in general people is panic. From a psychological standpoint, panic is an emotion or attitude that arises in response to threats [4]. In the COVID-19 pandemic, psychological panic has manifested in strong negative emotions such as anxiety and depression [5,6,7] and fear of being infected [8,9], which further disrupts daily life [10]. However, the risks of COVID-19 may not be equal across all groups. Lower-socioeconomic-status (SES) individuals who possess fewer resources suffer greater severity and mortality [11,12,13]. However, they also have methods to mitigate negative emotions, such as justifying the social system or depending on the authorities [14]. In this study, we examine the relationship between SES and psychological panic in inhabitants of China during the COVID-19 pandemic. The key to understanding this relationship lies in the role of trust in government, that is, a higher level of trust among people of lower SES reduces their psychological panic. However, this role might be weaker in people with higher authoritarian orientations who also had a high level of political trust before the pandemic [15]. Thus, this research mainly focuses on the effects of trust in government and an authoritarian personality on the psychological panic experienced by people of different socioeconomic status during the COVID-19 pandemic.

Socioeconomic status is defined as the relative position in an economic and social hierarchy, and it is typically combined by objective indicators of one’s access to resources [16]. The positive relationship between SES and mental health in ordinary life was confirmed in previous studies [17,18]. Theory from the rank-based perspective of social class suggests that individuals with lower status might develop certain patterns of thought as a result of their lived experiences at that lower rank; for instance, they tend to be more vigilant to threats and exhibit heightened reactions [19]. Furthermore, lower SES is related to higher risk perception [20] and more stress [21]. During the COVID-19 outbreak in Spain, education was found to be a negative predictor of perceived threat [22]. However, some studies have provided contradictory evidence. For example, SES had no substantial effect on individuals’ anxiety among Swiss university students [23]. A representative survey in China also found that personal and family income were not related to one’s sense of fear during the COVID-19 pandemic [24]. Although these results might be counterintuitive, they are consistent with viewpoints from system justification theory [14], which proposes that disadvantaged people may have increased support for government systems to help them defend against unpredictability in reality [25]. Given its nature, the pandemic is a highly politicized issue. People have depended on the government to provide valid information and guide their behaviors, and its effective action confirmed individuals’ certainty of not being infected [26]. Thus, trust in government might play a critical role in the link between SES and psychological panic.

Trust in government, as the most important part of political trust, speaks of citizens’ confidence and belief that the outcomes of government actions are consistent with their expectations [27]. Extensive research showed that political trust in China has been consistently high since 1995 [28,29], but there are SES differences: rural residents and people with lower levels of education have higher trust in government [30]. System justification theory also proposes that, even though lower-status individuals may lack control over the environment, they could compensate for personal control and deal with stress through belief in the existing system and trust in the government [31,32,33]. Meanwhile, lower-status individuals may feel it is impossible to escape the dangerous environment during the COVID-19 pandemic, so they do not resist the sociopolitical system, and they exhibit higher support to the government [34]. Thus, SES might be negatively related to trust in the government in China during the pandemic. Moreover, it positively impacts individuals’ mental health, such as perceived control and security [35]. Control/alienation theory states that people gain control over stress by seeking support from others [36], and government actions such as announcing information and instituting preventative measures during the pandemic can control the spread of the virus [37]. Thus, people who trust the government would have a sense of control over the pandemic and experience less psychological panic. Survey studies have also proven the relationship between political trust and mental health. For example, Cheung and Tse found that trust in the government negatively affected anxiety during the SARS crisis [38]. During the COVID-19 pandemic, a study also found that high political trust can ameliorate the impact of uncertainty and reduce the perceived threat [26]. Thus, higher trust in the government among lower-status individuals may lead to less psychological panic.

**Hypothesis** **1** **(H1).** *There were indirect relationships between SES and psychological panic through trust in the government during the COVID-19 pandemic in China, such that lower SES individuals have higher trust in government and thereby feel less psychological panic.*

**Figure 1:**
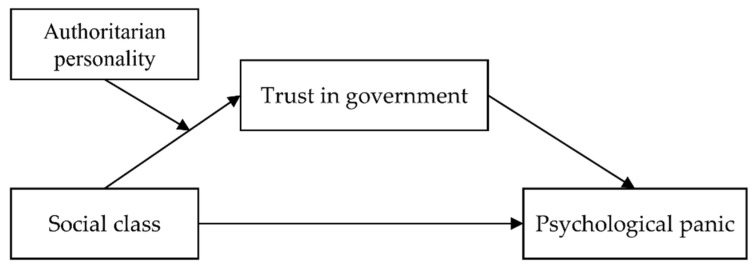
Hypothesized model.

Trust in the government is both psychological capital to help people obtain control and mitigate panic in a crisis, and an important political attitude in social life that could be influenced by personality factors [39]. These factors also play an important role in different-status individuals’ psychological responses to COVID-19 [40]. Thus, the indirect effect of trust in government between SES and panic during COVID-19 may also be affected by personality factors, one of which is an authoritarian personality. People with higher authoritarianism are more likely to be obedient toward authority and conform to traditional social norms [41], and this authoritarian orientation positively predicts political trust in East Asian societies [15]. The authoritarian personality might be an important moderator of the relation between SES and trust in government in China. Among people with low authoritarian personalities, individuals from disadvantaged backgrounds would continue to have more trust in the government than those with a higher status would. In a threat situation such as COVID-19, poor people who have fewer resources to protect themselves from the virus may be more dependent on powerful external forces [12], and trust in the government could help them to retain a sense of control in the pandemic. Meanwhile, higher-status individuals who have relatively sufficient access to information and medical resources have no need to put more trust in the government than usual. By contrast, for people with high authoritarian personalities who are characterized by authoritarian submission and conventionalism [40], the difference in trust in government between lower- and higher-status individuals might be smaller because political trust is higher among all of them [15]. Thus, the effect of social class on trust in the government, and the indirect effect of the latter on psychological panic, are moderated by authoritarian orientation. In other words, a low authoritarian personality could be a boundary condition of these effects. The moderated mediation model is shown in Figure 1.

**Hypothesis** **2 (H2).** *An authoritarian personality has moderated the effect of SES on trust in government during the COVID-19 pandemic in China, such that the effect is weaker when the authoritarian orientation is high.*

**Hypothesis** **3 (H3).** *An authoritarian personality has moderated the indirect effect of trust in government on the relationship between SES and psychological panic during the COVID-19 pandemic in China, such that the indirect effect is weaker when the authoritarian orientation is high.*

We focused on psychological panic in people of different socioeconomic status during the COVID-19 pandemic. The major goal was to test the indirect effect of trust in government between SES and psychological panic, and whether having an authoritarian personality could moderate this effect. Considering the rank differential pattern of “strong central, weak local” political trust in China [42], we separately examined the role of trust in central and local governments. 

## 2. Materials and Methods

### 2.1. Participants

This study was part of a national survey conducted in China between 11 and 12 February 2020, when COVID-19 had affected people across the country. The participants were 933 individuals (582 men and 351 women) aged between 17 and 61 years (M = 30.04, SD = 8.19). All participants were recruited from the Tencent Questionnaire, a Chinese online platform similar to Amazon’s Mechanical Turk. The sample size represented the largest number of participants recruited during the predetermined data phase. 

### 2.2. Materials

#### 2.2.1. Trust in Government

Participants completed two measures of trust in the central and local governments. Each questionnaire had three items [43]: “I trust the central (or local) government to fully consider the interests of the people”; “I trust that the central (or local) government has sufficient capacity to do a good job in pandemic prevention”; and “Although the central (or local) government has many shortcomings regarding the pandemic prevention work, I trust that they will continue to improve” (1 = completely disagree, 7 = completely agree). The score was calculated for each participant by taking the mean of their responses to the three items (α = 0.88 and 0.88).

#### 2.2.2. Socioeconomic Status

The SES index in health research mainly focuses on education level, occupation, and income [16]. Due to the complexity of the occupation concept [44] and the Chinese people’s fluctuating opinion on it [45], we did not select it for this study. Following previous research [46,47], SES was indexed by personal education and income in this study. Participants reported their highest level of education by choosing: (1) primary school, (2) middle school graduate, (3) high school graduate or equivalent education completed, (4) junior college graduate, (5) college graduate, or (6) postgraduate degree. The median score was college graduation. Participants reported their average monthly income by choosing: (1) <CNY 1000; (2) CNY 1000–CNY 2000; (3) CNY 2000–CNY 3000; (4) CNY 3000–CNY 5000; (5) CNY 5000–CNY 8000; (6) CNY 8000–CNY 12,000; (7) CNY 12,000–CNY 15,000; (8) CNY 15,000–CNY 20,000; (9) >CNY 20,000. The median was between CNY 5000 (USD 778) and CNY 8000 (USD 1245), which is higher than the per capita disposable income of CNY 2682 (USD 417) for residents in China in 2020 (https://data.stats.gov.cn/english/easyquery.htm?cn=C01 (accessed on 13 October 2021) The national per capita disposable income indicator was calculated by household and included non-income residents such as children. However, participants in this study reported their personal income; therefore, it was higher than this indicator). Correlation between the highest level of education and average monthly income was positive (r = 0.241, *p* < 0.001). We standardized each index and averaged the scores to compute an overall measure of SES.

#### 2.2.3. Psychological Panic

Psychological panic was divided into three types: state anxiety, perception of life disturbance, and intention to flee [48]. Participants were asked to answer the questions on a 7-point scale (1 = completely disagree, 7 = completely agree). State anxiety refers to the degree of anxiety about the pandemic, and six items were used [49]: “I feel tense”; “I feel upset”; “I feel worried”; “I feel calm” (reverse-scored); “I feel content” (reverse-scored); and “I feel relaxed” (reverse-scored). The perception of life disturbance had three items: “I cannot work and study at ease”; “I mind touching other people because it increases the chance of infection”; and “Compared with usual, I feel sick more often”. Intention to flee was measured by a single item: “When I know that the epidemic is spreading locally, I hope to go to a safer place”. The score was calculated for each participant by calculating the mean of their responses to the 10 items (α = 0.87). The items measuring psychological panic were used and validated in Chinese samples during the SARS crisis [48].

#### 2.2.4. Authoritarian Personality

Six items were used to measure participants’ authoritarian personalities. They were modified and validated in a Chinese sample in a previous study [50]: “Groups and troublemakers who challenge the authority and social order of the government must be severely punished”; “Our country needs a strong, determined leader who will crush evil and spread positive energy”; “Obeying and respecting authority are the most important virtues that children should learn”; “The leaders of the government are like parents of a big family, and everyone should obey them”; “It is important to respect our Confucian traditions and customs”; and “Even if the parents’ request is unreasonable, the children should follow it” (1 = completely disagree, 7 = completely agree). The score was calculated for each participant by calculating the mean of their responses to the six items (α = 0.76). 

#### 2.2.5. Risk Degree and Demographics

We used two items to measure the risk degree of participants: “How likely is COVID-19 to spread in your community/town?” and “To what extent does the pandemic threaten the safety of your and your family’s life?” (1 = not at all, 7 = very much, r = 0.44, *p* < 0.001; Cronbach’s α = 0.61). Participants were also asked to provide demographic information about their gender and age.

## 3. Results

### 3.1. Sociodemographic Statistics for Participants

Table 1 summarizes the broad sociodemographic patterns of the sample.

### 3.2. Preliminary Analyses

Descriptive statistics and correlations for all variables are presented in Table 2. Correlation analysis indicated that psychological panic and SES were negatively related to trust in local and central governments. An authoritarian personality was positively related to trust in local and central governments.

### 3.3. Mediation Analysis

**Figure 2:**
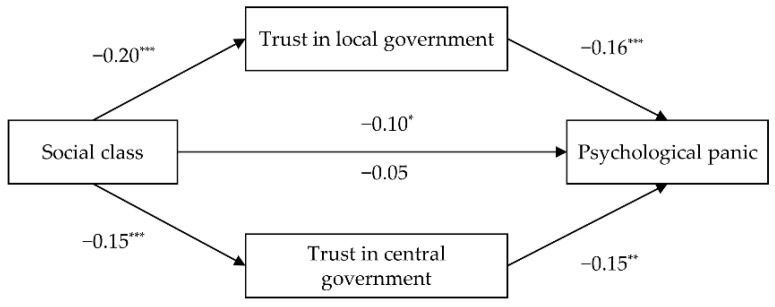
Mediating role of trust in government on the relationship between SES and psychological panic, controlling for risk degree, age, and gender (* *p* < 0.05, ** *p* < 0.01, *** *p* < 0.001).

We conducted mediation path analysis using the PROCESS procedure (Model 4) [51] to test the suppression effect of trust in the government. Bootstrapping was set to 5000 resamples. We included age, gender, and risk degree as covariates, as age and gender affect individuals’ mental health [52], and the severity of the pandemic varied in different districts of China [53]. Further, covariates were significantly associated with the dependent or mediation variables. As shown in Figure 2, after controlling for covariates, the indirect effect of SES on psychological panic through trust in local (B = 0.03, 95% CI = 0.01, 0.06, SE = 0.01) and central governments (B = 0.02, 95% CI = 0.006, 0.04, SE = 0.01) was significantly negative, yielding 95% CIs that did not contain 0. These indirect effects were still significant without the inclusion of covariates. Thus, Hypothesis 1 is supported. The direct effect of SES on psychological panic was significantly positive after removing the indirect effect, B = −0.10, 95% CI = −0.20, −0.006, SE = 0.05, yielding 95% CIs that did not contain 0. Thus, even though lower SES individuals feel more psychological panic, their higher trust in local and central governments leads to less psychological panic.

### 3.4. Moderated Mediation Analysis

**Figure 3:**
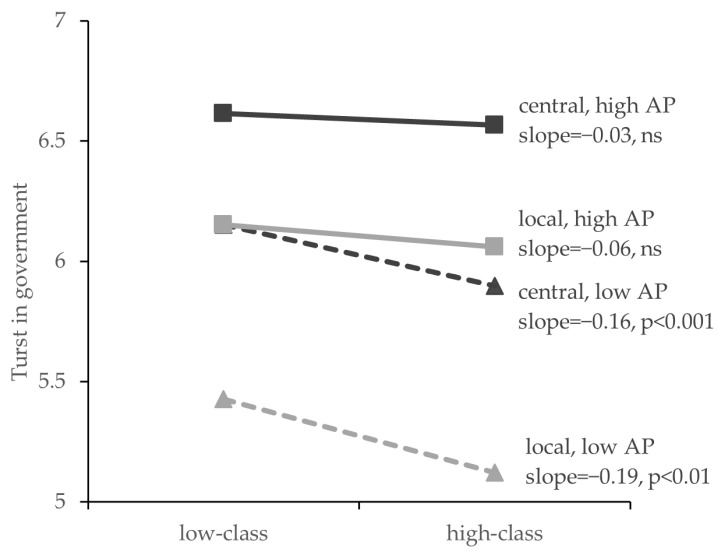
Moderating effect of authoritarian personality on the relationship between SES and trust in central and local government. Note: AP = authoritarian personality.

We conducted moderated mediation path analysis using the PROCESS procedure (Model 7) [51] to test the moderating role of the authoritarian personality. Bootstrapping was set to 5000 resamples. We also included age, gender, and risk degree as covariates, and the significances remained the same without the inclusion of covariates. Results in Table 3 show that the interaction effect on trust in the central government was significant. Figure 3 shows that there was a weaker negative relationship between SES and trust in central and local governments when the level of the authoritarian personality is high. Thus, Hypothesis 2 is supported.

Furthermore, as shown in Table 4, when authoritarian personality was lower, the indirect effects of trust in local and central governments were significant, and 95% CIs did not contain 0. However, when authoritarian personality was high, the indirect effects were not significant, and 95% CIs contained 0. Thus, Hypothesis 3 is supported.

## 4. Discussion

Although the impact of COVID-19 on individuals’ lives is not equally distributed among SES [11,12], studies observed inconsistent results on whether lower SES individuals feel more negative emotions. This complex effect could be because of the buffering influence of heightened political trust in lower SES individuals, which reduces psychological panic during the pandemic. Results are consistent with this hypothesis. Using a large sample during the COVID-19 outbreak in China, we found negative (direct) association between SES and psychological panic. However, this association was suppressed and nullified by trust in governments, which shared negative associations with SES and psychological panic. Furthermore, for people with high authoritarianism whose higher political trust provides a buffer against threats to mental health, this indirect effect of trust in government is weakened. 

Prior work indicated that people with lower SES are usually faced with more mental stress [54]. Meanwhile, several reports showed that political trust can mitigate negative emotions during a pandemic [26,38]. Our findings fit with and extend prior work by demonstrating that the direct negative relationship between SES and psychological panic fails to emerge due to the suppressing effects of political trust. This supports arguments from system justification theory, which states that the motivation to support and justify social systems could be stronger among disadvantaged groups [32], and this enhanced system justification serves a palliative function of making people feel better [14].

The moderating effects of authoritarian personality on the relationship between SES and trust in government is consistent with prior studies, which indicated that an authoritarian personality both promotes trust in government [15] and influences the effect of other factors on political trust [55]. For example, high respect for authority could weaken the relationship between the government’s economic performance and trust in government in China [56]. This result also highlights the importance of considering personality traits in exploring the relationship between social issues and individual mental health. 

Our study offers a novel perspective to explain the counterintuitive phenomenon that people with lower SES do not feel more panic regarding COVID-19 in China. Despite the evidence that disadvantaged people have suffered more in the pandemic [11,13], our finding indicates that they could manage their panic through trust in the power of government. Thus, results developed and connected the views of the rank-based perspective of social class and system justification theory, in that both the higher vigilance to threats [19] and the higher trust in government exhibited among lower SES individuals [25] simultaneously affect their level of psychological panic, but the two forces are confrontational. Higher trust in the government buffers lower SES individuals’ response to the pandemic, causing them to show less psychological panic. 

The results of this study also have practical implications for policy makers and mental-health professionals. First, the current work highlights the benefits of political trust in alleviating distress experienced by people with lower SES during the COVID-19 pandemic [57]. Thus, in addition to specific actions to improve the situation of disadvantaged groups during the pandemic, policy makers should also provide sufficient information about the government’s positive performance to build and retain lower SES citizens’ confidence and trust in the government, as this could mitigate their panic. Second, providing information about governments’ effective performance could also be a strategy for managing patients’ anxiety. Thus, psychiatrists should maintain a closer connection with governments or policy makers. Third, for people with diverse personality traits, different communication strategies should be employed. According to our results, it is necessary to increase political trust among those with low authoritarianism in the low SES group. Fourth, although people with lower status feel less panic related to political trust, direct effects indicate that they still suffer more mental distress than people with higher status do. However, the economic crisis might hinder efforts to obtain professional help; thus, mental-health services should be made more accessible to these people [58]. 

This study has some limitations. First, we did not examine the compensation role of personal control, which may be a factor, as control/alienation theory argues that people gain control by supporting others [36]. Future research is needed to further ascertain the impact of political trust on lower SES individuals’ psychological panic. Second, we did not measure participants’ occupation, which is an important indicator of SES and might be associated with both trust in the government and psychological panic during COVID-19. We did not measure participants’ mental health status or physical conditions, which might also be confounding variables associated with psychological panic. Third, while indirect effects were statistically significant, the effect sizes were small, and this may have been affected by the measurement method and sample quality. More importantly, it also implies that there may be other mediation mechanisms in the relationship between SES and psychological panic. Fourth, the present research only examined hypotheses in China, but the effect of political trust and the mechanism of psychological panic might be different in other cultures [59]. Future research could conduct a cross-cultural comparison of the relationship between SES and mental health during the pandemic.

## 5. Conclusions

This study showed that trust in government acts as a protective factor against the negative impact of SES on psychological panic during the COVID-19 pandemic in China, suggesting that lower-status individuals’ higher trust in government may buffer their psychological panic. However, the roles of trust in the government were disparate for people with higher or lower levels of authoritarianism in their personalities. Among people with low authoritarian personalities, individuals from disadvantaged groups exhibited higher levels of trust in government, and this trust further predicted less panic. However, for people with high authoritarian personalities, there was no difference between lower- and higher-status individuals’ trust in government. Our findings highlight that the increased threat to lower SES individuals’ mental health may be counteracted by their higher political trust, and personality factors may also play an important role in this process.

## Figures and Tables

**Table 1:** Sociodemographic statistics for the sample in our study (N = 933).

Variable	Categories	Frequency	Valid Percentages
Gender	Male	582	62.4%
	Female	351	37.6%
Age	17–24	251	26.9%
	25–30	345	37.0%
	31–40	236	25.3%
	41–50	77	8.3%
	51–61	24	2.6%
Educational attainment	Primary school or below	2	0.2%
	Middle school graduate	16	1.7%
	High school graduate or equivalent education completed	58	6.2%
	Junior college graduate	313	33.5%
	College graduate	442	47.4%
	Postgraduate degree	102	10.9%
Average Monthly Income	<CNY 1000	52	5.6%
	CNY 1000–CNY 2000	51	5.5%
	CNY 2000–CNY 3000	105	11.3%
	CNY 3000–CNY 5000	238	25.5%
	CNY 5000–CNY 8000	235	25.2%
	CNY 8000–CNY 12,000	143	15.3%
	CNY 12,000–CNY 15,000	47	5.0%
	CNY 15,000–CNY 20,000	35	3.8%
	>CNY 20,000	27	2.9%

**Table 2:** Descriptive statistics and correlations about all variables (N = 933).

	M	SD	1	2	3	4	5	6	7	8
1. Psychological panic	4.46	1.26								
2. Trust in local government	5.68	1.19	−0.25 **							
3. Trust in central government	6.30	0.84	−0.20 **	0.56 **						
4. SES	0.00	0.79	−0.01	−0.13 **	−0.12 **					
5. Authoritarian personality	4.57	1.14	−0.09 **	0.38 **	0.37 **	−0.13 **				
6. Risk degree	3.81	1.44	0.32 **	−0.16 **	−0.07 *	0.08 *	−0.07 *			
7. Age	30.04	8.19	−0.01	0.04	0.08 *	0.15 **	0.04	0.05		
8. Gender	—	—	0.07 *	−0.09 **	−0.08 *	−0.05	−0.15 **	0.05	−0.02	

Note. * *p* < 0.05, ** *p* < 0.01. Gender (0 = male, 1 = female).

**Table 3:** Results of regression analysis predicting trust in government and psychological panic (N = 933).

	Dependent Variables											
	Trust in Local Government				Trust in Central Government				Psychological Panic			
	B	95%CI	SE	t	B	95%CI	SE	t	B	95%CI	SE	t
Independent variable												
SES	−0.13	[−0.22, −0.03]	0.05	−2.71 **	−0.10	[−0.16, −0.03]	0.03	−2.90 **	−0.10	[−0.20, −0.01]	0.05	−2.08 *
AP	0.36	[0.30, 0.43]	0.03	11.29 ***	0.25	[0.20, 0.30]	0.02	10.78 ***				
SES × AP	0.06	[−0.02, 0.14]	0.04	1.51	0.06	[0.003, 0.11]	0.03	2.06 *				
Trust in local government									−0.16	[−0.23, −0.08]	0.04	−4.07 ***
Trust in central government									−0.15	[−0.26, −0.04]	0.05	−2.77 **
Covariates												
Age	0.01	[−0.002, 0.02]	0.004	1.48	0.01	[0.003, 0.02]	0.003	2.77 **	0.001	[−0.01, 0.01]	0.005	0.10
Gender	−0.09	[−0.24, 0.06]	0.07	−1.17	−0.06	[−0.16, 0.05]	0.05	−1.05	0.07	[−0.09, 0.22]	0.08	0.85
Risk degree	−0.11	[−0.16, −0.06]	0.02	−4.39 ***	−0.02	[−0.06, 0.01]	0.02	−1.39	0.26	[0.20, 0.31]	0.03	9.54 ***

Note. * *p* < 0.05, ** *p* < 0.01, *** *p* < 0.001, AP = authoritarian personality.

**Table 4:** Conditional indirect effect as a function of trust in government (N = 933).

Value of Authoritarian Personality	Mediator					
	Trust in Local Government			Trust in Central Government		
	Indirect Effect	Boot SE	Boot 95%CI	Indirect Effect	Boot SE	Boot 95%CI
−1 SD	0.031	0.01	[0.01, 0.06]	0.025	0.01	[0.004, 0.05]
M	0.020	0.01	[0.01, 0.04]	0.015	0.01	[0.002, 0.03]
1 SD	0.009	0.01	[−0.01, 0.03]	0.004	0.01	[−0.01, 0.02]

## Data Availability

Data will be provided if requested from the authors.
